# Personalized Deep Learning for Substance Use in Hawaii: Protocol for a Passive Sensing and Ecological Momentary Assessment Study

**DOI:** 10.2196/46493

**Published:** 2024-02-07

**Authors:** Yinan Sun, Ali Kargarandehkordi, Christopher Slade, Aditi Jaiswal, Gerald Busch, Anthony Guerrero, Kristina T Phillips, Peter Washington

**Affiliations:** 1 Department of Information and Computer Sciences University of Hawaii at Manoa Honolulu, HI United States; 2 Department of Psychiatry University of Hawaii at Manoa Honolulu, HI United States; 3 Center for Integrated Health Care Research, Kaiser Permanente Hawaii Honolulu, HI United States

**Keywords:** machine learning, precision health, Indigenous data sovereignty, substance use, personalized artificial intelligence, wearables, ecological momentary assessments, passive sensing, mobile phone

## Abstract

**Background:**

Artificial intelligence (AI)–powered digital therapies that detect methamphetamine cravings via consumer devices have the potential to reduce health care disparities by providing remote and accessible care solutions to communities with limited care solutions, such as Native Hawaiian, Filipino, and Pacific Islander communities. However, Native Hawaiian, Filipino, and Pacific Islander communities are understudied with respect to digital therapeutics and AI health sensing despite using technology at the same rates as other racial groups.

**Objective:**

In this study, we aimed to understand the feasibility of continuous remote digital monitoring and ecological momentary assessments in Native Hawaiian, Filipino, and Pacific Islander communities in Hawaii by curating a novel data set of longitudinal Fitbit (Fitbit Inc) biosignals with the corresponding craving and substance use labels. We also aimed to develop personalized AI models that predict methamphetamine craving events in real time using wearable sensor data.

**Methods:**

We will develop personalized AI and machine learning models for methamphetamine use and craving prediction in 40 individuals from Native Hawaiian, Filipino, and Pacific Islander communities by curating a novel data set of real-time Fitbit biosensor readings and the corresponding participant annotations (ie, raw self-reported substance use data) of their methamphetamine use and cravings. In the process of collecting this data set, we will gain insights into cultural and other human factors that can challenge the proper acquisition of precise annotations. With the resulting data set, we will use self-supervised learning AI approaches, which are a new family of machine learning methods that allows a neural network to be trained without labels by being optimized to make predictions about the data. The inputs to the proposed AI models are Fitbit biosensor readings, and the outputs are predictions of methamphetamine use or craving. This paradigm is gaining increased attention in AI for health care.

**Results:**

To date, more than 40 individuals have expressed interest in participating in the study, and we have successfully recruited our first 5 participants with minimal logistical challenges and proper compliance. Several logistical challenges that the research team has encountered so far and the related implications are discussed.

**Conclusions:**

We expect to develop models that significantly outperform traditional supervised methods by finetuning according to the data of a participant. Such methods will enable AI solutions that work with the limited data available from Native Hawaiian, Filipino, and Pacific Islander populations and that are inherently unbiased owing to their personalized nature. Such models can support future AI-powered digital therapeutics for substance abuse.

**International Registered Report Identifier (IRRID):**

DERR1-10.2196/46493

## Introduction

### Background

Methamphetamine abuse is highly prevalent in Hawaii, especially among Indigenous Pacific Peoples [1]. Since the 1980s, Hawaii has been considered the methamphetamine capital of the United States. Data from the Pacific Health Analytics Collaborative show that from 2015 to 2018, in total, 1.5% of Hawaiian residents used methamphetamine annually [2]. This was more than twice the national rate of 0.6%. According to the Bureau of Alcohol, Tobacco, Firearms, and Explosives, 71% of all drug cases in Hawaii were related to methamphetamine [3]. There are major methamphetamine-related disparities between Native Hawaiian, Filipino, and Pacific Islander individuals and people of other races in Hawaii, with Native Hawaiian, Filipino, and Pacific Islander individuals exhibiting elevated rates of illicit substance abuse [4]. According to the Centers for Disease Control and Prevention, Native Hawaiian and Pacific Islander high school students exhibited lifetime methamphetamine use of 7.7% versus 3.7% in White students, 2.7% in Black students, 3.1% in Asian students, and 5.7% in Hispanic and Latino students, and these disparities continued into adulthood [5]. Digital interventions powered by artificial intelligence (AI) have the potential to reduce these disparities by aiding clinicians in remotely providing care and monitoring patients between visits, especially among populations living in rural areas in Hawaii. Furthermore, such technology could be useful in relapse prevention for those hoping to maintain abstinence. In Hawaii in 2021, a total of 96% of residents possessed at least 1 piece of hardware with internet capacity, with only 4% lacking access to such equipment, indicating widespread internet access among the population [6].

AI-based detection of substance abuse using biometric signals measured by wearables is an active field of research across several research laboratories globally, as documented in a 2022 review paper by Rumbut et al [7]. Studies in this field tend to collect prediction labels through the remote administration of an ecological momentary assessment (EMA), a methodology in which participants are periodically asked to answer questions about their psychiatric or behavioral state while living as usual [8-10]. Notably, previous AI models suffered from clinically unacceptable performance. The primary reason for this lackluster performance is that prior methods attempted to train models using data from many patients, which is the status quo in deep learning because of the requirement of massive data sets for successful training. In contrast to these prior works, we will develop personalized AI models using a method developed in PW’s laboratory, which are capable of learning baseline patterns of human behavior and transferring this knowledge to prediction tasks with very few labeled examples to learn from.

Our research laboratory is particularly qualified to carry out this project as we are already developing computer science methods to support the personalization of AI models for large and mostly unlabeled data streams, with promising preliminary data and publications in preparation supporting this methodology. As a T1 (covering basic discovery) or T2 (initial human trials) project, our proposed study is significant both in terms of equitable substance abuse therapeutics and as a general methodology for clinical and translational research in other domains. There are countless situations in health care where vast amounts of unlabeled data are collected from a single patient. Annotations for the event of interest (eg, substance abuse) are frequently sparsely dispersed. The development of predictive supervised models is infeasible in such circumstances because classical approaches cannot handle the complexity of data, and modern deep learning approaches require vast amounts of data.

### Innovation

To support machine learning (ML) development in situations where vast longitudinal data are collected with minimal human-provided annotations, we propose the development of personalized ML models, which are trained solely on an individual’s unlabeled data to learn feature representations that are specific to their baseline temporal dynamics. We are creating a novel method and framework that has never been explored in health care, consisting of pretraining neural networks to learn the temporal dynamics of a patient’s biosignals. This method will enable deep networks to be trained using relatively small data sets, which would not be possible without the self-supervised approach proposed in this study. This technique is particularly well-suited for massive data sets with few labels.

The application of personalized AI to a diverse population of persons using substances is unique. Native Hawaiian, Filipino, and Pacific Islander communities have been understudied and could benefit from novel treatments to address methamphetamine use. Although we will apply this technological innovation toward the prediction of methamphetamine use, multimodal time-series personalization can be applied to a variety of other biological and health problems where (1) multiple signals are sparsely emitted, (2) the baseline signal patterns are specific to each individual, and (3) it is infeasible to acquire the vast amounts of labels required to train a supervised deep learning model.

This method has the potential to dramatically advance the field of precision health care by enabling reliable AI predictions from massive but mostly unlabeled data sets, which are trained in a self-supervised manner on data from a single user. This setting of large, unlabeled data sets with sparse supervision appears frequently in the field of digital health care. Notable examples include passive mobile sensing studies for mental health and well-being [11-20], digital therapeutics for children with autism spectrum disorder that record videos of the child [21-37], and passive brain sensors for brain-computer interfaces [38-46]. As such, this study protocol can be considered as one of the first tests of a broader emerging paradigm in precision health.

## Methods

### Overview

**Figure 1:**
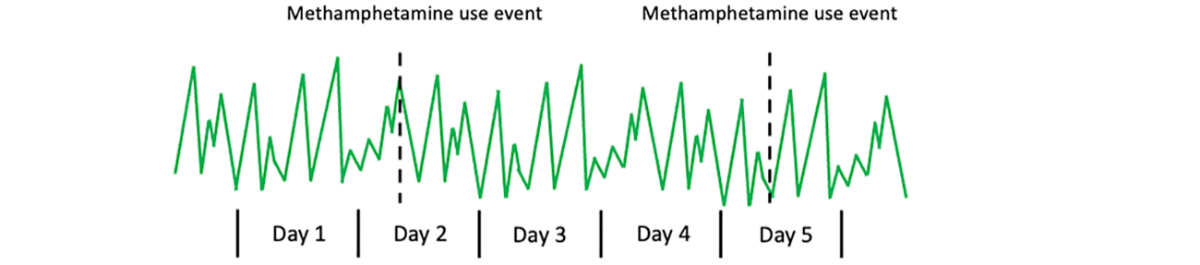
In many biomedical domains, there exist large data sets with sparse annotations of health events. We propose a “personalized self-supervised learning” method that can support the training of deep neural networks in such scenarios. We will evaluate our method primarily on the prediction of methamphetamine use events using biosignal data from a Fitbit device.

The long-term methodological goal of the proposed work is to develop novel AI methodologies for predicting health events (eg, methamphetamine use and cravings) using biosensors in a personalized manner. This technical innovation will be applied toward the detection of drug use events from wearable device sensors. The inputs to the proposed AI models are Fitbit (Fitbit Inc [47]) biosensor readings, and the outputs are predictions of methamphetamine use or craving (Figure 1). The methods developed can be applied to a variety of biomedical domains.

Diagnostic ML models are typically trained and deployed at a population level. In this traditional scenario, a single model is developed to make predictions for all individuals within a population. However, in several health contexts, an event of interest occurs repeatedly for an individual. For example, patients with diabetes have repeated blood glucose spikes, and chronically stressed individuals might have repeated blood pressure spikes. In these cases, ML models can be developed by conducting supervised training on the individual’s data only, resulting in a separate personalized model per individual. Although deep learning models have achieved state-of-the-art performance in a variety of health contexts, neural networks require massive data sets that are infeasible to collect for an individual. However, recent advances in self-supervised learning (SSL), or the subfield of ML focusing on pretraining models without any human-provided labels, have made it possible to realize the personalized ML diagnostics paradigm using deep learning by pretraining the weights of a neural network such that it can learn the baseline temporal dynamics without any labels. The pretrained model can then use transfer learning on relatively few labels that are acquired solely from the individual in question. This methodology can work particularly well in scenarios where massive amounts of unlabeled data are collected, such as with continuously worn devices.

### Aim 1: To Understand the Feasibility of Remote Digital Monitoring and EMAs in Native Hawaiian, Filipino, and Pacific Islander Communities by Curating Longitudinal Fitbit Biosignals With the Corresponding Substance Use and Craving Labels

#### Description

**Figure 2:**
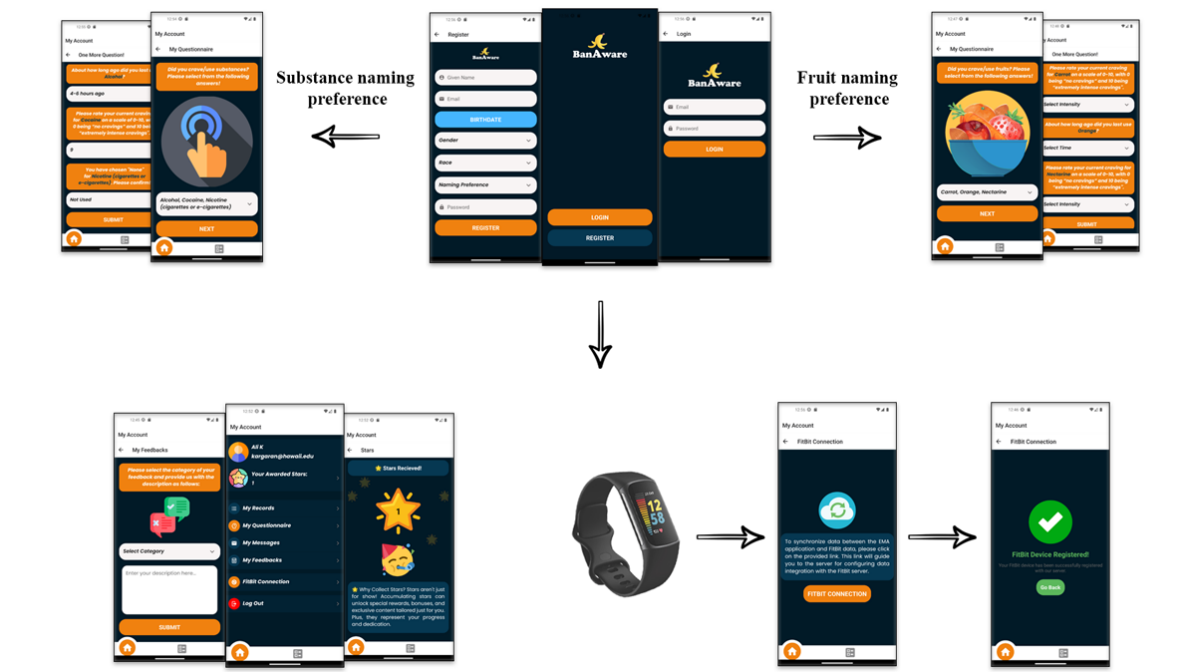
User interface of the ecological monitoring app provided to participants.

We will recruit 40 carefully selected Native Hawaiian, Filipino, and Pacific Islander participants who are either in treatment or have received services from one of our community partners to participate in a 4-week remote Fitbit data collection and concurrent EMA study. EMA studies, which involve periodic digital self-reports about psychiatric and behavioral outcomes *in the wild*, have often been used to understand substance abuse [48], including among persons who use methamphetamine [49]. We expect ≥80% complete data from approximately 25% (10/40) of the participants (refer to the *Recruitment* section for justification). Each participant will wear a study-provided Fitbit Charge 5 watch during all waking hours for at least 15 hours each day. Apart from wearing the device and periodically recording an EMA about their methamphetamine use via a mobile smartphone app (Figure 2), participants will be asked to follow their normal routine throughout the study.

#### Participant Recruitment and Management

We will recruit participants from a combination of sites, including the Hawaii Health and Harm Reduction Center and other sites where the clinical collaborators have connections (ie, Hina Mauka). Potential participants will be eligible for the study if they (1) are aged ≥18 years, (2) self-report consumption of methamphetamine on ≥2 different days per week on average, (3) have no plans to leave Oahu for at least 1 month, and (4) own a smartphone with either a data plan or regular access to a Wi-Fi connection. Potential participants will be excluded if they (1) are homicidal or suicidal, (2) cannot provide informed consent, (3) are not able to complete interviews in English, (4) are expecting incarceration or plan to leave Oahu within the next month, or (5) are unable to provide names and contact information for at least 2 verifiable locator persons for retention purposes.

We will recruit 40 participants in total. A secondary analysis of EMAs for methamphetamine abuse monitoring measured the percentage of participants who reached ≥80% compliance at different frequencies of methamphetamine use, finding that approximately 50% of persons who use methamphetamine 1 to 3 times per month met this 80% compliance bar, and 40% using 1 to 2 days per week met the bar, and 25% using 3 to 4 days per week met the bar [50]. Therefore, we anticipated that approximately 25% (10/40) of the participants will reach ≥80% compliance rate, which is sufficient to demonstrate the feasibility of our AI method, as a separate analysis will be conducted for each participant (ie, 1 model per participant).

#### Data Collection

We will leverage the existing application programming interface provided by Fitbit to record the user’s watch sensor readings and upload the data to the cloud. The Fitbit application programming interface provides access to heart rate (HR), gyroscope and accelerometer readings, breathing rate, blood oxygen saturation (SpO_2_) level, and skin temperature sensor readings. These biosensors have previously been used to predict substance abuse and cravings using AI [51-55]. The data will be managed on each participant’s smartphone device through an app, implemented for both iOS and Android, which we are actively developing. The study team will install the app on the user’s smartphone and configure the Fitbit device during study onboarding.

We will run the study with 8 (20%) of the 40 participants at a time as we have 8 Fitbit Charge 5 devices, resulting in 5 batches of data collection periods. We will record background characteristics, substance use, and treatment history during study intake, and we will record questions pertaining to the tolerability and obtrusiveness of the app during study outtake (Textbox 1). The smartphone app will record EMA responses from the participants throughout the 1-month study period (Textbox 1). At each EMA, we will ask participants to list the approximate times (eg, date, hour, and minute) of their methamphetamine intake in the past 24 hours via a user interface on the smartphone app. Participants will be asked to do the same for cravings. Participants will be prompted to provide EMA responses both when drastic signal changes are detected (ie, event-triggered EMA) and every 24 hours (ie, fixed-interval EMA).

**Study intake and outtake measures and ecological momentary assessment (EMA) questions collected from each participant.**

**Background characteristics (intake)**

- To better describe the sample, participants will be asked about their gender, sexual orientation, age, race or ethnicity, marital status, education, income, employment, housing, and health insurance status.

**Substance use and treatment history (intake)**

- Questions will be developed to assess current and history of substance use and participation in treatment services.

**Tolerability (study conclusion)**

- How comfortable was the device?

**Obtrusiveness (study conclusion)**

- Did the device change your daily routine? Do you have any concerns with continuously Fitbit usage?

**Methamphetamine use (EMA)**

- Have you used meth since the last time we contacted you?
- Approximately what time did you last use meth?
- How did you use meth the last time you used?

**Craving (EMA)**

- Please rate your current craving or desire to use methamphetamine at this exact moment on a scale of 0-10, with 0 being “no cravings” and 10 being “extremely intense cravings.”

We will institute procedures to reduce the burden associated with EMA and increase compliance as suggested by Burke et al [56]. To reduce the burden related to time commitment, we are compensating for every signal-contingent response and providing additional compensation when participants respond to >80% of prompts. Participants will be trained extensively on the EMA protocol at baseline, and if they experience any technology-related issues, our research assistants will help troubleshoot these issues remotely. Participants who experience technology-related problems will not have their compensation reduced because of missing prompts.

We will store the curated data from each participant (Figure 2) on a centralized server hosted on Amazon Web Services (AWS; Amazon). Data uploaded from both wearable systems and the smartphone will first run through a preprocessing server hosted on an elastic cloud computing (EC2) instance with data stored on DynamoDB (Amazon). Each table will have columns for the participant ID and timestamp. To ensure privacy and Health Insurance Portability and Accountability Act (HIPAA) compliance, we will encrypt all server-side data and require secret access keys for data access. DynamoDB tables are automatically encrypted on the server side. To add an additional layer of security, we will implement client-side encryption on the mobile app, ensuring encrypted data transmission across an https connection to move data between the devices and AWS. The data will not be accessible without a secret access key. All data will be anonymized.

An anonymized version of participant data will be made available to other computational researchers as a publicly available data set. This data set will be stored on AWS on a HIPAA-compliant server and will be password protected. Researchers will only gain access to this data set by signing a data use agreement. Such data sets exist for activity and emotion recognition from wearable data, but the prediction of methamphetamine use from these measurements will be a challenging task, and other ML practitioners can improve upon our initial AI models with the release of the deidentified data set. This will be the first publicly available data set that includes substance use self-reports alongside wearable sensor readings.

#### Data Analysis and Interpretation

We will measure the success rate of the remote data collection procedure using the response rate to EMA notifications. We hypothesize that we will observe higher compliance rates with event-triggered EMAs than fixed-interval EMAs. Furthermore, we will document qualitative challenges with the data collection process, tolerability, and unobtrusiveness (Textbox 1). We will conduct an interview with the participants at the study conclusion when the devices are returned. The research team will qualitatively code interview responses to derive recurring themes and design insights.

#### Potential Pitfalls and Mitigation Strategies

This analysis plan is uniquely robust to incomplete data collection because a separate AI model will be trained and evaluated for each participant. There is no requirement for equivalent data streams between participants nor will the analysis be prevented if the full 28-day data collection period is not achieved. The ML strategy can work with only a few logged methamphetamine use events. We expect approximately 25% (10/40) of the participants to complete the study at a sufficient level of compliance to support personalized ML analysis. This will provide sufficient data to demonstrate the feasibility of personalized ML analysis.

We have budgeted a 4-month buffer period beyond the 5 months required for complete data collection to account for participant delays and no-shows. Because participant data will be uploaded to AWS daily, we will remotely monitor participants through an automated tracking system already developed in our research laboratory and will cease the study if compliance is not logged after 4 days. Another possible issue is Fitbit theft or loss. To minimize this risk, participants will be compensated a minimum of US $135 for study completion and a maximum of US $210, which will be paid when the Fitbit device is returned. In the case of Fitbit device breakage or loss, our laboratory will purchase additional devices with funds separated from Center for Pacific Innovations, Knowledge, and Opportunities, up to a limit of 6 additional devices along the course of the study period.

### Aim 2: To Develop Real-Time Personalized AI Models Predicting Methamphetamine Use and Craving With Fitbit Sensor Data

#### Description

On the basis of extensive support from prior literature, we hypothesize that AI solutions can detect periods of both methamphetamine use and cravings with high sensitivity through the personalization of ML models. Such models will achieve high performance on a single individual through the finetuning of each model using only the data curated from the person of interest for model training. These personalized predictions can trigger the onset of digital therapy. We will develop two AI models per participant: (1) a model that detects methamphetamine use in real time and (2) a model that predicts methamphetamine craving in real time. We hypothesize that model personalization using novel self-supervised pretraining strategies will outperform traditional state-of-the-art AI techniques with <5% of the required label data.

#### ML Model Training

**Figure 3:**
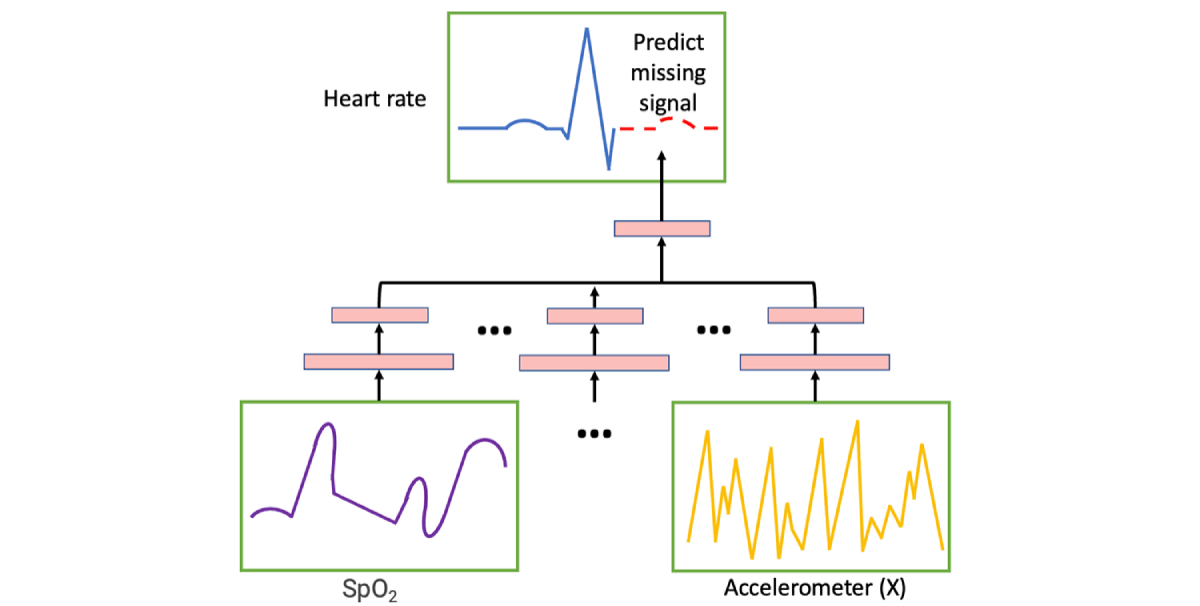
The key methodological computer science innovation of this protocol is the personalization of machine learning models that make predictions from biosignals time-series data without user-provided labels. In this figure, we depict a neural network that is trained to predict a heart rate signal given SpO_2_ levels and accelerometer signals from a single participant. SpO_2_: blood oxygen saturation.

The inputs to the models will consist of a separate 1D convolutional backbone pretrained for each biometric modality. The convolutional features will be fused upstream into a shared joint dense representation space and finally a dense prediction layer with linear activation for regression prediction (Figure 3). We will implement all models using TensorFlow (Google Brain) [57].

The data augmentation techniques that we apply to the signals will be domain specific, keeping in mind the inherent dynamics of each sensor. For example, for accelerometer data, rotations simulate different sensor placements, and cropping is used to diminish the dependency on event locations [58]. Across several modalities, sensor noise can be simulated through scaling, magnitude warping, and jittering [58]. We will be careful not to apply augmentation strategies that might change the meaning of the underlying signal.

#### Model Personalization

SSL is usually used to pretrain an entire data set with no explicit labeling by humans to guide the supervision task. We propose to redesign the SSL paradigm toward the task of model personalization. By pretraining a model only on the vast amounts of data curated from a single individual, the weights of the neural network will learn to make predictions using the inherent structure of each participant’s biosignals. This is essential because baseline HR, SpO_2_, skin temperature, and movement patterns, regardless of stress, will vary drastically across individuals, limiting the performance of general-purpose ML models.

**Figure 4:**
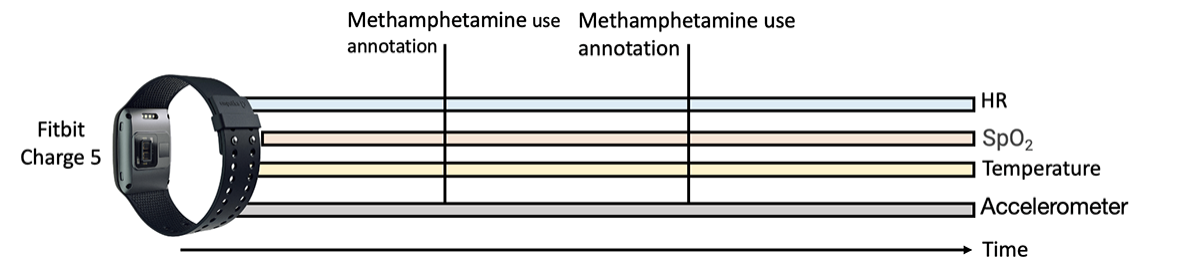
Depiction of the proposed data set, consisting of continuous Fitbit Charge 5 sensor readings and the corresponding methamphetamine use and craving annotations from 20 participants collected over a 4-week period. HR: heart rate; SpO_2_: blood oxygen saturation.

We plan to exploit the multimodal time-series nature of the collected data to perform novel SSL pretraining. We will use ≥1 signal to predict the value of another signal source (Figure 4). The motivation for this approach is that the biometrics of interest recorded by Fitbit are correlated [59-62].

#### Data Analysis and Interpretation

We will train the model on the first 75% of the data (by time) and calculate the balanced accuracy, precision, recall, *F*_1_-score, and area under the receiver operating characteristic curve for the final 25%. This evaluation pattern mimics real-world use, where a model will be calibrated by a user before real-world deployment. It is important to emphasize that we will train and test a separate personalized ML model for each individual (up to 40 separate models).

In a manner similar to our preliminary data, we will evaluate the models by comparing the performance with respect to the number of labeled examples used for supervised finetuning. A plot of this comparison will elucidate the number of methamphetamine annotations required for model calibration to an individual. We will create a separate plot for each study participant as the ML portion of this protocol tests the personalization of ML models rather than a general-purpose one-size-fits-all ML model, which is more typical in ML evaluations.

#### Feasibility

Self-supervised pretraining has been successfully demonstrated in several contexts in computer science and even health care [59,63,64], although not in the personalized context that we will explore, except for preliminary results that we have recently published [65-68]. Multimodal SSL has demonstrated success in prior literature [69-72], although not in the personalized manner in which we will innovate.

### Ethical Considerations

This protocol was approved by the University of Hawaii Institutional Review Board (protocol #2022-01030). In addition, this study has received further scrutiny and approval from the University of Hawaii Data Governance Process (request #230410-3).

Informed consent will be provided by the participants on paper during the intake session of the study.

Participants will provide information about their substance use on a smartphone app that we have created and will install on the phones of each participant. Because Fitbit is owned by Google, participants’ Fitbit data will be uploaded directly to Google’s cloud servers, which uses the same level of security as other Google products, such as Gmail.

Access to each participant’s Fitbit data on Google’s cloud servers is implemented through OAuth, which provides clients with secure delegated access to server resources on behalf of a resource owner (ie, the participants of this study). This mechanism is used by companies such as Amazon, Google, Facebook, Microsoft, and X (X Corp, formerly known as Twitter) to permit the users to share information about their accounts with third-party apps or websites. In this case, the “third party” is the study team.

Access to each participant’s annotations of substance use and craving from their smartphone app will be immediately uploaded to our secure and encrypted server on AWS, which is HIPAA compliant [73]. The participant’s data will be immediately removed from their smartphone after successful uploading to AWS.

All participant data will be analyzed on AWS. A fully anonymized version of the data set will be released to researchers who sign a data use agreement, which will be approved by the University of Hawaii Data Governance Office.

As a precaution, the interface on the app will not be labeled as “substance consumption” and “substance use” but rather as “banana consumption” and “banana use.” Furthermore, our data will be stored and labeled as “fruit” rather than “substance use.”

The participant’s data that will be accessible to the study team will include biometrics data from Fitbit, labels of “banana consumption” and “banana use” with the corresponding timestamps, and a unique participant ID. Digital data will only contain participant IDs rather than identifiable information. A paper copy of the participant’s mapping from the participant ID to the name and contact information will be stored on paper and securely locked in a lockbox hidden in PW’s desk. The lockbox in PW’s desk is secured with a key that only PW has access to, and his desk is in his office, which is secured with another key that only PW has access to. PW’s office is located within a suite of offices, which is secured by a third key that only professors in the Information and Computer Sciences Department have access to and which currently only PW has access to.

We will anonymize all the collected data. We will be provided with a Federal Certificate of Confidentiality from the National Institutes of Health, which will protect participants and assure confidentiality and privacy. KTP, a member of the mentorship and community teams, has found that having a Certificate of Confidentiality helps retain participants in lengthy projects.

### Compensation Type and Amount

We will provide the participants with US $135 for participation in the study. This amount is commensurate compensation for the requested work (wearing a smartwatch during all waking hours of the day for 4 weeks while continuously annotating their craving events). In addition, this compensation amount is above the market rate of a Fitbit device, helping to mitigate the risk of device theft by study participants.

US $20 will be provided to the participants to cover their transportation expenses for attending 2 in-person meetings on campus.

Furthermore, we will use snowball sampling as a form of recruitment, where participants can choose to refer their acquaintances to the study. Enrolled participants will be encouraged to refer other eligible participants to the study and will receive US $5 each for up to 3 referrals who enroll. The participants will be given 3 recruitment cards to distribute to eligible participants. When a new and eligible enrollee presents the card, the recruiting participant will receive US $5 compensation. If the recruiting participant has already completed the study, they will be contacted via their assigned study phone number (eg, by phone or SMS text messaging) or email to receive the compensation. If this proves unsuccessful, we will reach out to locator contacts or send a letter notifying the participant that they are eligible for the additional compensation.

Finally, we can compensate participants an extra US $40 as an incentive for providing responses on schedule and consistently throughout the study period.

## Results

Starting from November 2023, a total of 5 participants visited our laboratory and received Fitbit devices, including 4 (80%) male individuals and 1 (20%) female individual, aged between 22 and 63 years, representing 3 different ethnicities: 3 (60%) are White, 1 (20%) is Mexican, and 1 (20%) is Filipino Hawaiian.

Among the 5 participants, 178 logs have been collected. They completed an average of 8.6 days of EMA activity reporting, with each participant logging their data approximately 4 times per day. In total, the participants reported 40 instances of substance craving and 61 instances of substance use, including methamphetamine, alcohol, cannabis, and nicotine.

Fitbit devices recorded sensor data, including HR, number of steps, SpO_2_, HR variability, and breathing rate. HR and number of steps were tracked throughout the day, whereas SpO_2_ level, HR variability, and breathing rate were monitored during sleep.

Challenges related to EMA prompt reception were initially experienced by 1 (20%) of the 5 participants, but these were promptly resolved by the research team. In addition, labor-intensive reporting of simultaneous substance use and documenting of constant nicotine use posed difficulties for this participant. Another participant noted increased awareness of substance-related thoughts owing to EMA prompts.

## Discussion

### Preliminary Findings

To our knowledge, this is the first study to evaluate the feasibility of using a mobile app–based EMA to prospectively capture substance use among the Native Hawaiian, Filipino, and Pacific Islander population. Despite challenges, this study provides evidence to support the feasibility and acceptability of using EMA methods for collecting data on substance use in this population.

Following our research protocol, we successfully recruited 5 participants for our study in the first month of recruitment. Our research protocol, which included 4 scheduled prompts per day, was designed to represent a lower to moderate participant burden [74,75]. Each participant consistently provided an average of 4 logs per day. We received no significant issues or complaints from the participants, and their logging activities have been continuous. This partially aligns with prior studies by Phillips et al [76], Turner et al [50], and Hanson et al [77], who found that it is feasible and acceptable to use EMA to evaluate substances (eg, alcohol and methamphetamine) with people who come from historically marginalized groups.

Meanwhile, 1 (20%) of the 5 participants showed hesitancy regarding privacy concerns when discussing certain types of substances, as reported in the study by Han et al [78] that substance use can still be associated with social stigma. This suggests that building and maintaining a trusting relationship with the participants throughout the study, and even afterward, is crucial.

Despite concerns raised by Adams et al [79] about the potential impact of busy schedules on response rates, no significant problems were encountered in this regard as long as participants initiated their study. However, instances were experienced in which eligible individuals needed to reschedule their meetings because of their demanding work schedules. To accommodate their availability, the research team maintained a flexible schedule to encourage these individuals to visit the laboratory at their convenience. Weekly check-ins were conducted by the research team with the participants to ensure effective participation and promptly address any issues that arose.

Although prior studies, such as those by Cao et al [80] and Rodrigues et al [81], have successfully used Fitbit devices to collect sleep data, one challenge encountered by the research team is the absence of those data (eg, SpO_2_ level, HR variability, and breathing rate) that can only be collected while participants are asleep, despite the participants’ claims that they wear the Fitbit devices during sleep. To address this issue, the research team recommended that participants activate the sensitive mode for sleep sensitivity in the Fitbit app settings and ensure that they wear the devices tightly or close enough to their wrists. However, this recommendation might negatively affect the participants’ sleep quality, particularly for those who have self-reported sleeping problems.

This research protocol provides compensation to participants for reporting substance use events. However, this approach might lead to an increase in data noise, as observed when 1 (20%) participant provided 7 logs in a single day. The research team will diligently review these data to ensure their effectiveness and minimize potential noise.

None of the participants have reported participation exhaustion owing to the research protocol so far, despite such findings reported by Yang et al [75] and Semborski et al [82] in their studies. This may be attributed to the fact that many participants are still in their first or second weeks of the study. However, the research team observed that contacting participants >2 times per week could induce stress, particularly when technical issues persist without resolution. In response to these observations, the research team has currently limited their contact with each participant to a maximum of 2 times per week to mitigate participant exhaustion. Future studies may benefit from reducing contact frequency and providing clear and efficient instructions whenever such issues arise. Another issue related to participation fatigue or dropout is the extended waiting time for study entry. Despite the research team’s efforts to maintain contact with individuals who registered as early as May 2023, some of them may no longer be available or interested after several months of waiting. Future studies should minimize the waiting period to prevent potential disengagement.

One (20%) of the 5 participants expressed concerns about the increased contemplation of substances when responding to EMA prompts. Similarly, Fridberg et al [74] noted a slight increase in self-reported alcohol consequences in their EMA study. One plausible explanation is that certain EMA protocols may induce stress [82], potentially triggering substance use [83]. This study is in its initial stages and cannot provide any conclusions. The research team will closely monitor this participant and conduct weekly check-ins to ensure that there are no adverse effects.

This study has several limitations. Given the small sample size, the preliminary findings do not have enough statistical power to provide any significant findings on participants’ substance use and craving patterns or sociocultural factors that might affect those activities. The EMA prompts primarily collect quantitative data related to the scale, timing, or dates of substance use or cravings, excluding information on social and cultural factors that may trigger or influence substance cravings or use. For instance, we could not capture the presence of others during these moments using EMA prompts. Meanwhile, incorporating these questions into EMA prompts as self-reported data could have imposed cognitive demands on the participants, potentially impacting response rates and causing participation fatigue. To address this limitation, we plan to conduct in-depth semistructured interviews to explore the social and cultural factors.

One (20%) of the 5 participants highlighted the challenge of logging multiple substance use instances simultaneously and recommended the implementation of a more user-friendly feature in such situations. Currently, the research team is actively working to enhance this feature. In future studies, it is advisable to consider providing a more intuitive design when requiring participants to submit multiple logs in a more streamlined manner.

Our target population consisted of Native Hawaiian, Filipino, and Pacific Islander individuals; however, none of the team members belonged to this community, despite some team members having lived in Hawaii for >5 years. To address this limitation, the research team will work closely with the local partners, such as Hawaii Health and Harm Reduction Center and Hina Mauka, to ensure that key social and cultural factors are not overlooked.

Another limitation of our study is the exclusion of potentially eligible individuals who do not own a mobile phone or lack internet access. Future research endeavors could enhance inclusivity by considering the provision of mobile phones with a data plan for individuals who lack these resources.

### Conclusions

An EMA using smartphone apps offers a broad scope of research perspectives. Its capacity to capture phenomena instantaneously within real-life contexts grants the EMA a promising vantage point for understanding methamphetamine use and cravings among the Native Hawaiian, Filipino, and Pacific Islander population and other racial groups in Hawaii who have experienced methamphetamine-related disparities from the 1980s. Given the dearth of research on this issue within the targeted, historically marginalized group, sharing and presenting a standardized and innovative protocol for conducting EMA studies on methamphetamine use is crucial, which is the primary objective of this study.

We anticipate that this study will yield valuable insights into the feasibility of using EMA methods in this particular population and the sociocultural factors that can affect precise data acquisition. Furthermore, it will enable the development of personalized AI models for predicting methamphetamine-related behaviors within this demographic group. To date, our preliminary findings indicate promising outcomes associated with the use of EMA methods for data collection.

## Abbreviations

AI: artificial intelligence
AWS: Amazon Web Services
EMA: ecological momentary assessment
HIPAA: Health Insurance Portability and Accountability Act
HR: heart rate
ML: machine learning
SpO_2_: blood oxygen saturation
SSL: self-supervised learning
